# Cardiometabolic Risk Factors in Rosuvastatin-Treated Men with Mixed Dyslipidemia and Early-Onset Androgenic Alopecia

**DOI:** 10.3390/molecules26102844

**Published:** 2021-05-11

**Authors:** Robert Krysiak, Marcin Basiak, Bogusław Okopień

**Affiliations:** Department of Internal Medicine and Clinical Pharmacology, Medical University of Silesia, 40-752 Katowice, Poland; mbasiak@sum.edu.pl (M.B.); mbkdokop@mp.pl (B.O.)

**Keywords:** androgenetic alopecia, 3-hydroxy-3-methylglutaryl coenzyme A reductase inhibitors, mixed dyslipidemia, risk factors

## Abstract

Men with early-onset androgenetic alopecia are characterized by hormonal profiles similar to those observed in women with polycystic ovary syndrome. The purpose of this research was to investigate levels of cardiometabolic risk factors in 3-hydroxy-3-methylglutaryl coenzyme A (HMG-CoA)-treated men with early-onset androgenic alopecia. We studied two matched rosuvastatin-treated groups of men with mixed dyslipidemia: subjects with early-onset androgenic alopecia (group A) and subjects with normal hair growth (group B). Plasma lipids, glucose homeostasis markers, and levels of sex hormones, uric acid, hsCRP, homocysteine, fibrinogen, and 25-hydroxyvitamin D were measured before entering the study and six months later. Both groups differed in insulin sensitivity and levels of calculated bioavailable testosterone, dehydroepiandrosterone-sulfate, uric acid, hsCRP, fibrinogen, and 25-hydroxyvitamin D. Though observed in both study groups, treatment-induced reductions in total cholesterol, LDL cholesterol, hsCRP, and fibrinogen were more pronounced in group B than group A. Moreover, only in group A did rosuvastatin deteriorate insulin sensitivity, and only in group B did the drug affect uric acid, homocysteine, and 25-hydroxyvitamin D. The impact of rosuvastatin on cardiometabolic risk factors correlated with insulin sensitivity, calculated bioavailable testosterone, and dehydroepiandrosterone-sulfate. The obtained results suggest that men with early-onset androgenic alopecia may benefit to a lesser degree from rosuvastatin treatment than their peers.

## 1. Introduction

The presence of polycystic ovary syndrome (PCOS) in women is associated with an increased arterial stiffness, increased carotid intima-media thickness, endothelial dysfunction, thrombotic complications, cerebrovascular events, and possibly also cardiovascular events [1]. Compared with brothers of healthy women, brothers of women with PCOS are characterized by increased levels of two-hours post-challenge glucose, increased values of insulin resistance markers, and higher values of systolic blood pressure, as well as higher prevalences of impaired glucose tolerance, metabolic syndrome, and type 2 diabetes [2,3,4,5,6,7,8]. Brothers of PCOS probands are also characterized by elevated levels of dehydroepiandrosterone-sulfate (DHEA-S) levels and lower concentrations of bioavailable testosterone [5,6,7]. Therefore, it is reasonable to assume that male relatives of PCOS probands should be screened, identified, and appropriately treated [9,10].

Interestingly, a very similar hormonal profile to that observed in male siblings of women with PCOS was reported in men with early-onset androgenic alopecia. The prevalence of androgenic alopecia increases with age, and it is estimated that up 30% of Caucasian men have androgenic alopecia by the age of 30, and 80% of Caucasian men are affected by this disorder in the course of their life [11,12]. The classic male-pattern of hair loss involves the temporal and vertex region while leaving a rim of hair at the sides and sparing the occipital region [12]. Male pattern hair loss probably results from both genetic susceptibility and excessive androgen action [13]. The estimated heritability of early-onset androgenic alopecia in men is about 80%, while genetic variability in the androgen receptor gene and ectodysplasin A2 receptor gene is a prerequisite for the development of this disorder [9,13]. Compared with control subjects, men with early androgenetic alopecia have shown increased levels of testosterone, DHEA-S, LH, and prolactin; decreased levels of FSH and sex hormone-binding globulin; and increased values of the free androgen index and the LH/FSH ratio [14]. In comparison with healthy subjects, the risk of metabolic syndrome was found to be 2.3 times higher in men with early-onset androgenetic alopecia [15]. There is also evidence that early-onset androgenic alopecia is associated with obesity, insulin resistance, dyslipidemia, hypertension, and atherosclerosis [10]. Genetic, hormonal, and metabolic similarities to PCOS mean that early-onset androgenic alopecia is regarded as a male equivalent of PCOS [16].

The results of a recent study indicated that cardiometabolic effects of atorvastatin are less pronounced in brothers of women with polycystic ovary syndrome than in the male siblings of unaffected women [17]. Moreover, high-dose treatment with rosuvastatin, the latest and the most potent statin that is currently available on the market and frequently used in clinical practice, was found to reduce circulating testosterone levels but not to affect levels of DHEA-S in men with coronary artery disease [18]. Because no previous study has investigated statin action in subjects with male-pattern hair loss, the purpose of this study was to investigate whether early-onset androgenic alopecia determines the impact of rosuvastatin therapy on cardiometabolic risk factors in men with mixed dyslipidemia.

## 2. Results

At study entry, there were no differences between both groups in age, smoking, body mass index, and blood pressure, as well as in the plasma levels of total cholesterol, LDL cholesterol, HDL cholesterol, triglycerides, glucose, estradiol, and homocysteine. Both groups differed in HOMA1-IR and levels of calculated bioavailable testosterone, DHEA-S, uric acid, hsCRP, and fibrinogen, which were higher in group A than in group B, as well as in 25-hydroxyvitamin D, which was higher in group B than in group A (Table 1 and Table 2).

Rosuvastatin did not result in serious adverse events, and all patients completed the study. Body mass index and blood pressure remained at a similar level throughout the study.

In both study groups, rosuvastatin decreased the levels of total cholesterol, LDL-cholesterol, hsCRP, and fibrinogen. Treatment-induced changes in total and LDL cholesterol, hsCRP, and fibrinogen were more pronounced in group B than group A. Rosuvastatin increased HOMA1-IR exclusively in group A, while the drug only reduced levels of triglycerides, uric acid and homocysteine and increased levels of HDL cholesterol, and 25-hydroxyvitamin D only in group B. Rosuvastatin treatment did not affect glucose, DHEA-S, total testosterone, bioavailable testosterone, estradiol, and the estimated glomerular filtration rate. After six months of treatment, there were differences between both groups in total cholesterol, LDL cholesterol, HOMA1-IR, DHEA-S, calculated bioavailable testosterone, uric acid, hsCRP, homocysteine, fibrinogen, and 25-hydroxyvitamin (Table 2).

At entry, total cholesterol and LDL cholesterol correlated with levels of uric acid, hsCRP, homocysteine, and fibrinogen (group A: r values between 0.24 (*p* = 0.0499) and 0.40 (*p* = 0.0011); group B: r values between 0.30 (*p* = 0.0345) and 0.47 (*p* = 0.0001)), and there were inversely correlated with levels of 25-hydroxyvitamin D (group A: r = −0.32 (*p* = 0.0285) and r = −0.41 (*p* = 0.0007); group B: r = −0.35 (*p* = 0.0122) and r = −0.44 (*p* = 0.0002)). Moreover, there were positive correlations between HDL cholesterol and 25-hydroxyvitamin D (group A: r = 0.48 (*p* < 0.0001); group B: r = 0.50 (*p* < 0.0001), triglycerides or HOMA1-IR and hsCRP and fibrinogen (group A: r values between 0.26 (*p* = 0.0385) and 0.47 (*p* = 0.0001); group B: r values between 0.29 (*p* = 0.0403) and 0.49 (*p* = 0.0001)), and there were inverse correlations between triglycerides or HOMA1-IR and 25-hydroxyvitamin D (group A: r = −0.35 (*p* = 0.0071) and r = −0.43 (*p* = 0.0008); group B: r = −0.41 (*p* = 0.0014) and r = −0.49 (*p* < 0.0001)), as well as between HDL cholesterol and hsCRP and fibrinogen (group A: r = −0.34 (*p* = 0.0087) and r = −0.40 (*p* = 0.0025); group B: r = −0.39 (*p* = 0.0037) and r = −0.47 (*p* = 0.0001)). Treatment-induced changes in uric acid, hsCRP, fibrinogen, homocysteine, and 25-hydroxyvitamin D inversely correlated with calculated bioavailable testosterone levels (group A: r values between −0.32 (*p* = 0.0298) and −0.42 (*p* = 0.0006); group B: r values between −0.35 (*p* = 0.0281) and −0.48 (*p* < 0.0001)) and DHEA-S (group A: r values between -0.24 (*p* = 0.0488) and −0.37 (*p* = 0.0046); group B: r values between −0.31 (*p* = 0.0011) and −0.47 (*p* < 0.0001)). All other correlations were not significant.

## 3. Discussion

In comparison with the control subjects, men with androgenic alopecia had increased plasma concentrations of DHEA-S and increased levels of calculated bioavailable testosterone. Because of the exclusion criteria and selection procedure, these findings could not be attributed to differences in body mass index, blood pressure, plasma lipids, concomitant disorders, or drug interactions. The hormonal profile of individuals with early-onset alopecia differed from that observed in the male siblings of PCOS probands, in whom elevated concentrations of DHEA-S coexisted with lower levels of calculated bioavailable testosterone [17]. Unlike bioavailable testosterone, in both studies, mean total testosterone levels were similar to those observed in control groups. This discrepancy may be explained by the fact that bioavailable testosterone (denoting the sum of the free and free weakly bound testosterone) calculated by Vermeulen’s formula (used in the current study) correlates with free testosterone levels when assessed by equilibrium dialysis [19,20]. Because only unbound testosterone binds the androgen receptor in target tissues in order to exert its activity, the obtained results seem clinically relevant. Under physiological conditions, DHEA-S is converted to testosterone by three enzymes: steroid sulfatase, 3β-hydroxysteroid dehydrogenase, and 17β-hydroxysteroid dehydrogenase type 3 [21,22]. Therefore, it is possible that in brothers of PCOS women, but not in men with early-onset androgenic alopecia, the activity of at least one these enzymes is slightly disturbed. In addition to increased levels of HOMA1-IR, uric acid, and hsCRP and a decreased concentration of 25-hydroxyvitamin D, as observed in both brothers of PCOS probands and men with early-onset alopecia, individuals with early-onset alopecia were characterized by elevated levels of fibrinogen. Interestingly, fibrinogen levels were found to correlate with the incidence rates of myocardial infarction and stroke and with cardiovascular mortality, while the risk of coronary artery disease in individuals with hyperfibrinogenemia was comparable to or higher than that in subjects with elevated total cholesterol levels [23]. Based on the obtained results, at least three conclusions may be drawn. Firstly, from the phenotype point of view, men with early-onset androgenic alopecia and male siblings of women with PCOS represent different clinical entities or constitute different spectra of the same entity. Secondly, they may differ in cardiometabolic risk, which seems to be higher in subjects with alopecia. Finally, because men with early-onset androgenic alopecia were more insulin-resistant than body mass index-, plasma lipid- and blood pressure-matched peers, this state may predispose subjects to type 2 diabetes, metabolic syndrome, and other conditions associated with insulin resistance.

The current study also showed that subjects with alopecia differed from the control group in the impact of rosuvastatin on lipids, insulin sensitivity, and levels of cardiometabolic risk factors. Individuals with early-onset alopecia were characterized by less pronounced changes in plasma lipids and cardiometabolic risk factors, as well as by a potentially unfavorable effect on insulin sensitivity. These findings indicate that men with early-onset male-pattern hair loss may benefit to a lesser degree from rosuvastatin therapy than subjects with normal hair growth, at least in the primary prevention of cardiovascular disease. This observation is of clinical significance because early-onset androgenic alopecia can be easily diagnosed based on anamnesis and basic clinical signs.

There are different possible explanations for dimorphism in rosuvastatin action between both study groups. Some our observations seemed to indicate that they are probably partially related to differences in insulin sensitivity. Both groups differed in baseline HOMA1-IR and in the impact of rosuvastatin on HOMA1-IR, present only in men with alopecia. Moreover, treatment-induced changes in circulating levels of uric acid, hsCRP, fibrinogen, homocysteine, and 25-hydroxyvitamin D levels correlated with HOMA1-IR. If this explanation is accurate, non-pharmacological treatment or metformin may restore the pleiotropic effects of rosuvastatin in subjects with early-onset androgenic alopecia, and we intend to verify this hypothesis in our future studies. Despite numerous clinical cardiovascular benefits of 3-hydroxy-3-methylglutaryl coenzyme A (HMG-CoA) reductase inhibitors in type 2 diabetes, statin therapy is associated with a slightly increased risk of developing new-onset diabetes [24]. Considering the results of our study, this risk may be higher in men with early-onset androgenic alopecia than in other groups of patients with indications for treatment with HMG-CoA reductase inhibitors.

According to another explanation, dimorphism in the lipid-lowering and extra-lipid effects of statins may be associated with differences in the baseline levels of endogenous androgens, particularly testosterone. Over the entire study period, the levels of DHEA-S and calculated bioavailable testosterone were higher in subjects with early-onset androgenic alopecia than in individuals without it, while the impact of rosuvastatin on all assessed cardiometabolic risk factors correlated with calculated bioavailable testosterone and, though to a lesser extent, with DHEA-S. Because HMG-CoA reductase inhibitors do not affect androgen levels [18], except for high-dose statin therapy, between-group differences in the impact of rosuvastatin cannot be attributed to their effect on plasma androgens. They are also not associated with differences in the conversion of androgens to estrogens because estradiol levels were similar in both study groups. It is much more probable that statins and testosterone may interact at the level of conversion of HMG-CoA to mevalonate. The pleiotropic properties of statins result from their inhibitory effect on the biosynthesis of some isoprenoids, particularly farnesyl pyrophosphate and geranylgeranyl pyrophosphate, that play essential roles in the posttranslational modification of numerous key proteins that act as molecular switches (especially Ras, Rac, and Rho) [25]. This mechanism may be attenuated by high testosterone levels. Even a single dose of exogenous testosterone was found to induce the expression of HMG-CoA reductase [26]. Moreover, testosterone has recently been shown to increase levels of key enzymes of the mevalonate pathway, and its high levels may stimulate the posttranslational prenylation and farnesylation of numerous small signaling proteins [27].

This study had some study limitations that require consideration. The study population exceeded the required sample size, but due to a relatively small number of participants, our findings should be verified in a large prospective study. We did not investigate hard clinical endpoints, such as morbidity and mortality rates. The study design did not allow us to conclude whether the obtained results may be explained by a “class effect” of HMG-CoA reductase inhibitors or only characterize rosuvastatin or some statins. Finally, because the study included only men with normal glucose homeostasis or prediabetes, it cannot be totally ruled out that the impact of rosuvastatin is different in individuals with coexisting type 2 diabetes.

In conclusion, compared with age-, body mass index-, and blood pressure-matched subjects with isolated mixed dyslipidemia, men with early-onset androgenic alopecia were characterized by impaired insulin sensitivity; elevated levels of DHEA-S, calculated bioavailable testosterone, uric acid, hsCRP, and fibrinogen; and lower levels of 25-hydroxyvitamin D. In addition to less expressed lipid-lowering properties, men with early-onset androgenic alopecia were characterized by less pronounced cardiometabolic effects of rosuvastatin. Contrary to the control subjects, the rosuvastatin treatment of men with alopecia deteriorated insulin sensitivity. The obtained results suggest that men with early-onset androgenic alopecia may benefit from rosuvastatin treatment to a lesser degree than other subjects with mixed dyslipidemia.

## 4. Materials and Methods

The study was conducted in accordance with the 1964 Helsinki Declaration and its later amendments. The experimental protocol was reviewed and approved by the Institutional Review Board. All participants provided written informed consent prior to enrolment in the study.

### 4.1. Patients

We studied two groups of young men (aged 18–35 years) with mixed dyslipidemia, defined as total cholesterol levels above 200 mg/dL, low-density lipoprotein (LDL) cholesterol levels above 115 mg/dL, and triglyceride levels of more than 150 mg/dL, despite complying with the lifestyle modification for more than 3 months before entering the study. Group A included 25 individuals with early-onset androgenic alopecia, while group B (which was a control group) included 25 matched men with isolated mixed dyslipidemia. Early-onset androgenic alopecia was defined as grade 3 vertex or more alopecia on the Hamilton and Norwood scale, which is used to classify the stages of male pattern baldness as diagnosed before the age of 30 years [28,29].

The study population was selected among a larger group of subjects with mixed dyslipidemia (*n* = 112), diagnosed and treated in our department. A power calculation using 80% power and a type I error of 0.05 indicated that at least 23 individuals would need to be enrolled in each group to detect a 20% difference in the effects of treatment on the measured cardiometabolic risk factors between the groups. The selection procedure, performed using the freely available PEPI-for-Windows computer program, was intended to obtain two groups matched for age, blood pressure, and body mass index. To minimize the impact of seasonal fluctuations in the study outcomes, similar numbers of participants were included in January or February (*n* = 26) and in July and August (*n* = 24).

The exclusion criteria were as follows: cardiovascular disease (with the exception of mild arterial hypertension), impaired renal or hepatic function, diabetes, thyroid disorders or other endocrine disorders, acute and chronic inflammatory processes, malabsorption syndromes, any other serious disorder, and any pharmacotherapy.

### 4.2. Study Design

Rosuvastatin was administered at a dose of 10 mg once daily at bedtime for 6 months. All participants were also given detailed advice about how to achieve the goals of lifestyle modification, which were a total fat intake of less than 30% of total energy intake, a saturated fat intake of less than 7% of energy consumed, a cholesterol intake of less than 200 mg per day, and an increase in fiber intake to 15 g per 1000 kcal. They were also recommended to do at least 150 min of moderate-intensity aerobic physical activity per week, as well as muscle-strengthening activities that were of moderate intensity and involved all major muscle groups on two or more days a week. Medication adherence was assessed every 6 weeks by means of a four-item Morisky–Green test and by pill count.

### 4.3. Laboratory Assays

All laboratory assays were carried out at baseline and 6 months later. Venous blood samples for laboratory were collected between 7.00 and 8.00 a.m. after 12-h overnight fasting in a quiet and air-conditioned room (constant temperature of 23–24 °C), and they were assessed in duplicate. Standard laboratory techniques were used to measure plasma glucose levels, plasma lipids (total cholesterol, LDL cholesterol, HDL cholesterol, and triglycerides), uric acid, and creatinine (Roche Diagnostics, Basel, Switzerland). The plasma levels of insulin, DHEA-S, total testosterone, estradiol, sex-hormone binding globulin, homocysteine, and 25-hydroxyvitamin D were assayed by direct chemiluminescence using acridinium ester technology (ADVIA Centaur XP Immunoassay System, Siemens Healthcare Diagnostics, Munich, Germany). The plasma levels of high-sensitivity C-reactive protein (hsCRP) were measured using an immunoassay with chemiluminescent detection (Immulite 2000XPi, Siemens Healthcare, Warsaw, Poland), while fibrinogen levels were measured by the Clauss technique using an automated BCS XP analyzer (Siemens Healthcare, Warsaw, Poland). The homeostasis model assessment 1 of insulin resistance index (HOMA1-IR) was calculated by multiplying plasma insulin (mIU/L) by plasma glucose (mg/dL) and dividing that by 405. Bioavailable testosterone was calculated on the basis of total testosterone and sex hormone-binding globulin levels using a freely available online calculator (www.issam.ch/freetesto.htm). The estimated glomerular filtration rate was calculated using the Modification of Diet in Renal Disease equation.

### 4.4. Statistical Analysis

Outcome variables were log-transformed for analysis to obtain a better approximation of the normal distribution, and they were transformed back for reporting in the tables. All analyses were adjusted for age, smoking, body mass index, and blood pressure as potential confounding factors. Both groups and differences between percent changes from baseline after adjustment for baseline values (reflecting rosuvastatin action) were compared by Student’s *t*-tests for independent samples. The differences between the means of variables within the same group were analyzed using Student’s paired *t*-test. Qualitative data were compared using the χ2 test. Correlations were assessed using Pearson’s correlation coefficient (r). Two-tailed *p*-values corrected for multiple testing below 0.05 were considered statistically significant.

## Figures and Tables

**Table 1:** Baseline characteristics of patients.

Variable	Group A ^a^	Group B ^b^	*p*-Value (Group A vs. Group B)
Number (n)	25	25	-
Age (years; mean (SD))	30 (5)	31 (5)	0.4829
Smokers (%)	32	28	-
Body mass index (kg/m^2^; mean (SD))	28.9 (4.3)	28.7 (4.6)	0.8745
Systolic blood pressure (mmHg; mean (SD))	131 (14)	129 (15)	0.62828
Systolic blood pressure (mmHg; mean (SD))	85 (6)	84 (6)	0.5585

^a^ Men with early-onset androgenic alopecia. ^b^ Control men.

**Table 2:** The effect of rosuvastatin on plasma lipids, glucose homeostasis markers, hormones, and the investigated cardiometabolic risk factors in young men with mixed dyslipidemia.

Variable	Group A ^a^	Group B ^b^	*p*-Value
			(Group A vs. Group B)
Total cholesterol (mg/dL; mean (SD))			
At the beginning of the study	262 (32)	267 (35)	0.6005
At the end of the study	216 (28)	196 (25) *	0.0105
*p*-value (post-treatment vs. baseline)	<0.0001	<0.0001	-
LDL cholesterol (mg/dL; mean (SD))			
At the beginning of the study	167 (23)	171 (26)	0.5672
At the end of the study	124 (20)	103 (15) *	0.0001
*p*-value (post-treatment vs. baseline)	<0.0001	<0.0001	-
HDL cholesterol (mg/dL; mean (SD))			
At the beginning of the study	43 (7)	42 (7)	0.6158
At the end of the study	46 (8)	48 (8) *	0.3812
*p*-value (post-treatment vs. baseline)	0.1647	0.0069	-
Triglycerides (mg/dL; mean (SD))			
At the beginning of the study	240 (62)	252 (58)	0.4832
At the end of the study	218 (51)	195 (43) *	0.0912
*p*-value (post-treatment vs. baseline)	0.177	0.0003	-
Glucose (mg/dl; mean (SD))			
At the beginning of the study	95 (10)	93 (12)	0.5251
At the end of the study	97 (11)	93 (8)	0.148
*p*-value (post-treatment vs. baseline)	0.5044	1	-
HOMA1-IR (mean (SD))			
At the beginning of the study	3.4 (0.7)	3.0 (0.6)	0.035
At the end of the study	3.9 (0.8) *	2.8 (0.7)	< 0.0001
*p*-value (post-treatment vs. baseline)	0.0228	0.2835	-
DHEA-S (µmol/L; mean (SD))			
At the beginning of the study	4.8 (0.9)	4.1 (1.0)	0.0123
At the end of the study	5.0 (1.2)	4.2 (1.1)	0.0177
*p*-value (post-treatment vs. baseline)	0.5082	0.7381	-
Total testosterone (nmol/L; mean (SD))			
At the beginning of the study	20.8 (6.4)	17.9 (6.0)	0.1049
At the end of the study	20.4 (7.2)	16.8 (5.8)	0.0574
*p*-value (post-treatment vs. baseline)	0.8364	0.5131	-
Calculated bioavailable testosterone			
(nmol/L; mean (SD))	8.09 (2.11)	6.31 (1.98)	0.0035
At the beginning of the study	8.26 (2.28)	6.02 (2.06)	0.0007
At the end of the study	0.7856	0.6141	-
*p*-value (post-treatment vs. baseline)			
Estradiol (pmol/L; mean (SD))			
At the beginning of the study	140 (29)	148 (32)	0.359
At the end of the study	135 (25)	143 (34)	0.348
*p*-value (post-treatment vs. baseline)	0.5169	0.5982	-
Uric acid (mg/dL; mean (SD))			
At the beginning of the study	4.9 (1.3)	4.2 (1.0)	0.038
At the end of the study	4.7 (1.2)	3.6 (1.0) *	0.001
*p*-value (post-treatment vs. baseline)	0.1641	0.0391	-
hsCRP (mg/L; mean (SD))			
At the beginning of the study	3.4 (0.9)	2.8 (0.8)	0.0162
At the end of the study	2.8 (0.9)	1.8 (0.7) *	0.0001
*p*-value (post-treatment vs. baseline)	0.0225	< 0.0001	-
Fibrinogen (mg/dL; mean (SD))			
At the beginning of the study	375 (82)	329 (64)	0.0318
At the end of the study	324 (70)	260 (59) *	0.001
*p*-value (post-treatment vs. baseline)	0.0221	0.0002	-
Homocysteine (μmol/L; mean (SD))			
At the beginning of the study	24 (8)	25 (9)	0.6798
At the end of the study	20 (7)	15 (6) *	0.0093
*p*-value (post-treatment vs. baseline)	0.0661	<0.0001	-
25-hydroxyvitamin D (ng/mL; mean (SD))			
At the beginning of the study	25 (10)	32 (10)	0.0169
At the end of the study	27 (8)	38 (9) *	<0.0001
*p*-value (post-treatment vs. baseline)	0.6446	0.0305	-
Estimated glomerular filtration rate (ml/min/1.73 m^2^; mean (SD))			
At the beginning of the study	92 (15)	93 (16)	0.8206
At the end of the study	92 (13)	94 (14)	0.6031
*p*-value (post-treatment vs. baseline)	1	0.8151	-

^a^ Men with early-onset androgenic alopecia. ^b^ Control men. * The impact of rosuvastatin (percent changes from baseline after adjustment for baseline values) stronger than in the second group.

## Data Availability

The data that support the findings of this study are available from the corresponding author upon reasonable request.

## Sample Availability

Samples of the compounds are not available from authors.

## Abbreviations

DHEA-S: dehydroepiandrosterone-sulfate; HDL: high-density lipoproteins; HMG-CoA: 3-hydroxy-3-methylglutaryl coenzyme A; FSH: follicle-stimulating hormone; HOMA1-IR: the homeostasis model assessment 1 of insulin resistance index; hsCRP: high sensitivity C-reactive protein; LDL: low-density lipoproteins; LH: luteinizing hormone; PCOS: polycystic ovary syndrome; SD: standard deviation
